# Pb Neurotoxicity: Neuropsychological Effects of Lead Toxicity

**DOI:** 10.1155/2014/840547

**Published:** 2014-01-02

**Authors:** Lisa H. Mason, Jordan P. Harp, Dong Y. Han

**Affiliations:** ^1^Department of Neurology, University of Kentucky College of Medicine, 740 S. Limestone, Lexington, KY 40536, USA; ^2^Department of Psychology, University of Kentucky College of Arts and Sciences, 106b Kastle Hall, Lexington, KY 40506, USA

## Abstract

Neurotoxicity is a term used to describe neurophysiological changes caused by exposure to toxic agents. Such exposure can result in neurocognitive symptoms and/or psychiatric disturbances. Common toxic agents include heavy metals, drugs, organophosphates, bacterial, and animal neurotoxins. Among heavy metal exposures, lead exposure is one of the most common exposures that can lead to significant neuropsychological and functional decline in humans. In this review, neurotoxic lead exposure's pathophysiology, etiology, and epidemiology are explored. In addition, commonly associated neuropsychological difficulties in intelligence, memory, executive functioning, attention, processing speed, language, visuospatial skills, motor skills, and affect/mood are explored.

## 1. Introduction

Neurotoxicity describes neurophysiological changes caused by exposure to toxic agents, which may result in cognitive changes, memory disorders, and changes in mood or onset of psychiatric disturbances (for review refer to Han et al., Caban-Holt et al., and Mason et al.) [1–3]. Common toxic agents include certain heavy metals, drugs, organophosphates, bacterial, and animal neurotoxins [4]. Each toxic agent results in unique presentations, depending on what neurophysiological changes occur following exposure. Furthermore, toxic exposure can be differentiated by acute exposure versus chronic exposure, with each type further affecting symptom presentation and outcomes. Depending on exposure type, neurotoxic exposure may result in central-nervous system damage, affective disturbances, and/or neurocognitive disruptions.

Neurotoxicity from heavy metals, including lead, mercury, and arsenic to name a few, is most commonly studied in two groups: chronic or acute exposure. Acute exposure often involves rapid onset of nausea, headaches, cognitive changes, and emotional disruptions. However, heavy metal exposure is often encountered in industrial workplace environments, where chronic, prolonged exposure is more likely. In such chronic exposure, neurodegeneration and psychiatric manifestations are more prevalent. Psychiatric manifestations may include increased depression, anxiety, and irritability. Chronic exposure may also result in prolonged and variable symptom presentations, including fatigue, decreased processing speed, fine and gross motor deficits, and generally decreased cognitive functioning [4]. The remainder of this review focuses on neurotoxicity following lead exposure. Pathophysiology, etiology, and epidemiology are explored, along with commonly associated neuropsychological difficulties.

## 2. Pb Toxicity 

### 2.1. Pathophysiology

*Plumbum* (Pb), a chemical element in the carbon group otherwise known as lead, is a soft and malleable metal, which is considered a heavy metal. Lead introduced into the bloodstream is excreted in urine and bile at a clearance rate of 1 to 3 mL/min, with a half-life of roughly 30 days. The remaining lead binds to red blood cells, is distributed throughout the soft tissues of the body, and eventually accumulates in bone. The half-life of bone-deposited lead ranges from 20 to 30 years. Turnover of bone tissue releases lead back into the bloodstream, and such processes as pregnancy, menopause, or lactation may increase blood lead levels by speeding bone tissue turnover. Lead in the body is measured with both blood and bone levels. Blood lead levels are more reflective of acute exposure, whereas bone lead levels better reflect cumulative exposure over time [5].

The presence of lead in the human body causes damage to the nervous system through several mechanisms. Direct effects on the nervous system may be classified as either morphological or pharmacological [6]. Morphological effects alter the development of the nervous system, particularly from the prenatal period through childhood. Such effects include disruption of key molecules during neuronal migration and differentiation [7]; interference with synapse formation, mediated by a reduction in neuronal sialic acid production [8]; and premature differentiation of glial cells [9].

Pharmacological effects result from the action of lead as a pharmacological agent in the CNS. Lead substitutes for calcium and, to a lesser extent, zinc and inappropriately triggers processes reliant on calmodulin [10]. Lead also interferes with neurotransmitter release, disrupting the function of GABAergic, dopaminergic, and cholinergic systems as well as inhibiting NMDA-ion channels during the neonatal period [11, 12]. *In vitro* studies have shown that lead activates protein kinase C in capillary cells and inhibits Na+/K+-ATPase in the cell membrane, interfering with energy metabolism [13, 14]. Within the cell, lead appears to interfere with calcium release from the mitochondria [14], resulting in formation of reactive oxygen species, speeding mitochondrial self-destruction through formation of the permeability transition pore, and priming activation of programmed cell death processes [15].

Indirect effects on the nervous system result from interference with other body systems that support nervous system function. Lead exposure has been found to increase risk of numerous conditions that may have adverse effects on nervous system function, including hypertension, impaired renal function, impaired thyroid function, vitamin D deficiency, and preterm birth [5]. There has been some debate as to whether lead exposure affects peripheral nerve conduction velocity, with some reviews concluding a lack of toxic effect [16] and others finding significant population-based changes [17]. The literature appears, at best, to support consistent subclinical lead effects on nerve conduction velocity [18].

The most severe neurological effect of lead exposure is lead encephalopathy [19], a response to very high doses of lead that results in development of irritability, headache, mental dullness and attention difficulty, memory loss, tremor, and hallucinations within weeks of exposure. Symptoms abruptly worsen to paralysis, convulsions, delirium, coma, or death. Children may develop lead encephalopathy at lower doses of lead than adults. Postmortem pathological findings include edema, capillary disruption, proliferation of glia, and diffuse anoxic injury [17].

### 2.2. Etiology

Acute high-dose exposure to lead is not the only source of lead-based neurotoxicity. Acute low-dose exposure also appears to produce measurable, if less dramatic, effects on nervous system function. Epidemiological studies have failed to find evidence of a threshold for neurological effects; recent large-scale, prospective studies suggest that blood lead levels below 10 *μ*g/dL significantly worsen intellectual functioning in children and that the strength of association is stronger at the low range of exposure [20–22].

Chronic exposure to environmental lead also has measurable effects on the nervous system due to the propensity for lead to accumulate in the bone over time. For instance, in an MRI study of 532 former lead workers, high tibia lead was associated with reduced total brain volume, lower volume of gray matter in the insula and cingulum, and diminished white matter volume in the parietal lobes [23]. Historically, the main sources of lead exposure were leaded gasoline, lead-based paint and plumbing, and solders used in food packaging. Leaded gasoline was thoroughly phased out of use by 1986 and is no longer a means of exposure. Similarly, lead solder in food cans was banned by the FDA in 1995. Lead plumbing still exists in buildings erected before 1940, and lead paint, though banned in the United States in 1978, is still found in some older buildings. These two latter sources perhaps explain the frequent association of lead exposure with low socioeconomic status [5, 24, 25].

Occupational lead exposure continues to be a source of both acute and chronic exposure, resulting in blood levels of 40 to 120 *μ*g/dL among participants in case studies and small cohort studies reviewed by the Agency for Toxic Substance and Disease Registry [5]. Some evidence suggests that effects of chronic exposure may persist long after exposure has ended, which is further discussed in Section 4 of this paper.

Prenatal exposure presents an additional risk for lead neurotoxicity. Maternal exposure to lead and overall maternal body burden of lead are closely associated with lead levels in the fetus, likely because lead appears to cross the placenta freely and because pregnancy increases systemic demand for calcium, resulting in higher bone turnover and consequent lead release into the bloodstream [26, 27]. Animal studies of brain lead content have demonstrated that the blood-brain barrier is particularly ineffective against lead in the prenatal stage, becoming more effective during weaning and even more so after weaning [28, 29].

Poor nutrition appears to increase risk of toxic effects of lead when exposure is held constant. Deficiencies in calcium, iron, and zinc have been specifically identified as risk factors. Calcium deficiency appears to increase both retention of lead and the severity of its toxic effects [30]. Low intake of dietary iron has similar effects and is perhaps more important because of relatively high risk of iron deficiency in childhood [31, 32]. At least one large-scale longitudinal study has shown that iron deficiency worsens the effects of lead exposure on neurobehavioral measures and blood cell production in infants, children, and pregnant women [33]. Zinc deficiency appears to result in a vicious cycle in that it increases lead absorption, which in turn increases zinc excretion [34]. The role of nutrition perhaps accounts for the finding that low socioeconomic status increases risk for persistence of cognitive deficits after prenatal lead exposure [35–37].

### 2.3. Epidemiology

The National Health and Nutrition Examination Survey (NHANES) estimated the prevalence of high blood lead levels (>10 *μ*g/dL) among US children to be 8.6% in the 1988–1991 time period and 1.4% in the 1999–2004 time period, representing an 84% decline. Moreover, the distribution of blood lead levels among children exhibited a shift toward lower levels; the majority of US children are no longer in the range of concern for risk of toxicity [24]. These results suggest that public health reforms targeting lead exposure were largely successful.

The incidence of lead poisoning is associated with numerous factors, including socioeconomic status, rurality, race, age, and the date one's residence was built. Inner-city poor children are at the highest risk, presumably due to the presence of lead in older building materials and reduced access to sources of nutrition. NHANES findings from the 1997 survey estimated that 16.4% of children in large cities and homes built before 1946 had high blood lead levels. Analyzed according to racial background, African American children appear to be at the highest risk, followed by Mexican American children, and then European American children [24]. Younger children appeared to be at additional risk, with about 9% of children 1–5 years old in the range of concern. Increased blood lead has also been associated with living in a rural area, though research on this factor is limited [38].

The Center for Disease Control and Prevention has identified 25 *μ*g/dL as the lower bound for the range of concern for adult blood lead levels. Incidence of concerning levels among adults almost halved from 1994 to 2007, falling from 14.0/100,000 to 7.8/100,000 [39]. According to the same study, occupational exposure was the primary source of lead exposure for adults, particularly in the mining and battery manufacturing industries. Risk was found to be higher among men than women because of occupational differences; there is no such gender difference consistently found among children. Epidemiological research on international populations is currently very limited.

## 3. Neuropsychology of Pb Toxicity

Lead exposure has effects on neuropsychological functioning that vary across the lifespan. Prospective studies have found that prenatal exposure, as measured by lead levels in umbilical cord blood, predicted slower development in the sensorimotor and visuomotor domains, as measured by the Bayley Scales of Infant Development [40, 41]. Numerous studies of children have shown that lead exposure reduces overall cognitive functioning in children, both cross-sectionally and longitudinally, but most such studies examine omnibus measures of intellectual functioning rather than domain-specific effects [35, 42–44]. In adulthood, it is apparent that chronic exposure to lead is more harmful to cognition than acute exposures. In a sample of demographically diverse, primarily middle-aged US adults, bone lead levels predicted worse cognitive performance in several domains, whereas blood lead level did not [45]. Researches on domain-specific cognitive effects are presented below.

### 3.1. Intelligence

Lowered intellectual scores have been most commonly noted in children following lead exposure. In reviewing pediatric cross-sectional studies on intellectual deficits following lead exposure, 3-point decrements in IQ have been noted when blood lead concentration increased from 5 to 20 *μ*g/dL and 5.3-point decrements in IQ when blood lead concentration increased from 5 to 50 *μ*g/dL [46]. Overall evaluation of the studies revealed a fairly consistent association between 1- and 3-point decrements when blood lead concentrations increased from 10 to 20 *μ*g/dL [46, 47]. These results suggest a dose-dependent decrease in intellectual ability following lead exposure, with higher lead exposure producing larger point decreases. While less consistently noted than pediatric effects, some decreased intellectual abilities have also been suggested in adults. The World Health Organization Program for Chemical Safety Task Group on Effects of Inorganic Lead [48] reviewed all extant literature and concluded that blood levels below 25 *μ*g/dL can reduce intellectual functioning in humans, with every 10 *μ*g/dL increment predicting an IQ decrease of 1–5 points. Further supporting a dose-dependent effect on intelligence in adults, increased levels of occupational lead exposure have been associated with lower overall cognitive scores and intelligence scores [49]. While initial studies of the cognitive effects following lead exposure focused on overall cognitive or intellectual effects, more recent research suggests the importance of examining domain-specific effects following lead exposure.

### 3.2. Memory

Some research has demonstrated lowered learning and memory scores in occupational lead-exposed adults [49, 50]. These results suggest that lead exposure is particularly detrimental in older adults, with individuals of age 55 and older producing lower learning and memory scores, among other cognitive declines. Despite this vulnerability in older adults, decreased memory performances have also been noted in adults younger than 55 years of age who have been exposed to high levels of lead. These individuals demonstrated decreased performances on verbal memory and visual memory tasks following lead exposure [50]. Lower visuospatial memory scores have been consistently documented [49], suggesting that lead exposure disrupts visuospatial skills and the ability to remember visual stimuli. Occupational lead exposure is also associated with lowered visual memory scores, specifically delayed recall of a complex figure [51]. Lowered verbal memory scores have also been noted following lead exposure, resulting in worsened immediate recall, delayed recall, and recognition. Not only does chronic exposure appear to affect both verbal and nonverbal memories, but it also appears to produce progressive decline. In this sample, both verbal and nonverbal memory tests scores further declined in subsequent years. This suggests that progressive memory decline may ensue for years following prolonged exposure [51].

### 3.3. Executive Functioning and Attention

Several studies have demonstrated declines in executive functioning following occupational lead exposure. Decreased executive functioning abilities on switching and inhibition tasks (Trails Making Test B and Stroop Task, resp.) have also been noted in a group comprised of individuals with a peak lead exposure of 20 *μ*g/g (tibia bone lead measurement) [51]. Lowered executive functioning scores were also found in earlier research using similar assessments and scores [52]. Unfortunately, much of the research related to executive functioning is confounded by a visual-motor component. Thus, the lowered executive functioning scores may also reflect and/or be exacerbated by processing speed or motor difficulties, discussed in following sections. Overall, the evidence is mixed regarding specific executive functioning deficits following lead exposure.

### 3.4. Processing Speed

Processing speed deficits following lead exposure have been noted, with results suggesting a dose-dependent relationship. Individuals exposed to high levels of lead have shown slowed decision-making abilities and reaction times. For instance, significant exposure-related decrements in decision-making speed and increased gaps in a detection/reaction time task have been noted [46]. These results also demonstrated subtle deficits in classification speed and accuracy in a category search task. These deficits were only noted in individuals with blood lead concentrations of 40 *μ*g/dL or more. A follow-up study with the same participants and testing battery confirmed this dose-dependency in neurobehavioral deficits [53]. However, both studies noted the main finding to be slowed sensory motor reaction time, which may have artificially lowered overall processing speed.

### 3.5. Language

In the language domain, the literature has consistently identified effects of lead exposure on comprehension and reading ability. In children, lead exposure has been associated with impaired verbal concept formation [54], poor grammatical reasoning [55], and poor command following on both standardized and *in situ* tasks [56]. Grammatical reasoning difficulty was also associated with lead exposure in a study of adult lead workers [53, 57]. The NHANES survey of 4,853 US children found significant effects of blood lead level on reading ability [58], as did a study of 501 children in Scotland [59]. The New England Children's Amalgam Trial (NECAL) found that moderate blood levels of lead (5–10 *μ*g/dL) predicted lower vocabulary performance among 534 children of ages 6–10 from urban Massachusetts and rural Maine [20]. A study of older men similarly demonstrated blood lead effects on the ability to define words and also found an effect on visual naming of contour drawings [60]. Non-word repetition deficits were associated with bone lead level in a sample of adolescent boys [56], but the task has not been repeated with other age groups.

### 3.6. Visuospatial Skills

The visuospatial domain of cognition appears to be particularly vulnerable to effects of lead toxicity. In adults of age 55 and older, higher bone lead level was associated with worse visuospatial function, as measured with visual reproductions, embedded figures, and block design tasks, and predicted further more decline in that domain relative to nonexposed controls after a 22-year delay [49]. A meta-analytic review of 22 studies of lead workers concluded that high blood lead levels are associated with significant effects on a block design task [61]. Another meta-analysis of 22 studies failed to replicate these results but did find significant effects on a task of speeded visual discrimination. In the Normative Aging Study, 141 older male participants were grouped according to blood lead level and by bone lead level [60]. Those with higher blood lead level required more time to produce accurate visual comparisons. Those with high blood and bone lead levels produced poorer copies of geometric figures and recalled visual patterns more poorly. In one study of adults, perceptual speed appeared to be affected by chronic lead exposure in a dose-dependent manner [62]. Blood lead level has been associated with poorer visuomotor integration in studies of Yugoslavian and primarily African American children [63–67] and, in one sample, blood lead levels measured in the sixth year of life predicted poorer visual construction at age 17 [67]. Neurologic examination uncovered impaired size discrimination and visual pursuit in a sample of Andean children as well [68].

### 3.7. Motor Skills

Lead exposure has long been known to disturb motor function. Particularly, visuomotor coordination impairment has been reported in studies of adult lead workers [54, 69–71] and lead-exposed children [55]. Manual dexterity has been demonstrated to suffer in a dose-dependent manner in lead workers from Republic of Korea and Venezuela [52, 62]. Moreover, baseline blood and bone lead levels in chronically exposed workers predicted the extent of further decline in manual dexterity over the course of 5 years [52]. In addition, the effects on visuomotor integration, studies of Yugoslavian and urban African American children found blood lead level to predict significantly poorer fine motor skills [63–66]. Gross motor speed, as measured by a finger tapping task, appears to be affected in lead-exposed children as well [55, 68].

### 3.8. Affect

Lead-related changes in affect are less researched than other functional domains. A study of 526 older adults in the Normative Aging Study found that anxiety, depression, and phobia were positively correlated with bone lead level [72]. Lead workers with high blood lead levels have been reported to experience greater interpersonal conflict than their less-exposed counterparts [73], and a review of 14 studies of low-level lead exposure concluded that lead-related interpersonal problems may be mediated by irritability and fatigue [74].

There is also growing evidence of early lead exposure linking to increased frequency of antisocial behavior including violent behavior [75]. A number of studies are revealing that antisocial tendencies like violent and aggressive behaviors correlate with environmental stressors like lead or polychlorinated biphenyls, beyond that of socioeconomic factors [76]. A 2010 study of 173 Brazilian adolescents with high level of surface dental enamel related lead exposure revealed a link with antisocial behavior, calling out for public policies to prevent lead poisoning [77].

## 4. Outcome 

Outcome following toxic lead exposure is dependent on several factors, including duration, amount of exposure, and age. Acute, high-level exposure and chronic low-level exposure each have differing outcomes. While individuals with asymptomatic lead exposure demonstrate the best outcomes, those who become symptomatic after exposure to acute high levels of lead often also demonstrate good outcomes. High-level exposure presents initially as lethargy and later progresses to coma and seizures. Death is rare if exposure is expediently and appropriately treated, but may occur if increased cranial pressure secondary to encephalopathy is prolonged [78]. As discussed later, nephropathy due to acute lead exposure may be reversible in some cases. Individuals exposed to chronic low levels of lead are likely to exhibit worse prognosis, with the potential for progressive renal decline, lower sperm counts or impotence in males, increases in miscarriage and smaller babies in females, and increased depression, aggression, and other affective disorders [78]. Despite these potentially serious consequences, other than some motor difficulties and increased affective disorders, most lead exposure is not likely to produce long-term cognitive changes in adults [78].

However, there is some evidence that occupational lead exposure may continue to cause progressive cognitive deficits years after exposure [49, 79]. In a sample of 535 former lead workers, bone lead levels measured 16 years after last exposure were associated with poorer cognitive performance [80] and predicted progressive decline [51, 79]. Some research also suggests increased vulnerability for cognitive difficulties in older populations, with high levels of bone lead predicting lower cognitive performances in individuals over the age of 55 [49]. Finally, pediatric exposure has been shown to have far more drastic consequences than adult exposure. Pediatric lead exposure often results in more severe neurological damage, as demonstrated by associations between exposure and long-term difficulties with learning and overall intelligence [46, 78].

## 5. Neurorehabilitation

The primary focus of treatment for lead exposure is to reduce the level of circulating lead in the bloodstream. To that end, the first-line intervention for lead poisoning is to cease lead exposure and administer chelation therapy. Chelation involves introduction of one or more agents that bind with lead and facilitate its excretion. Recommended chelation agents include oral meso-2,3-dimercaptosuccinic acid (DMSA) and intravenous or intramuscular ethylenediaminetetraacetic acid (EDTA). Encephalopathic individuals with severe, acute lead toxicity are usually treated with intramuscular dimercaprol, either alone or in combination with EDTA. Both cessation of lead exposure and chelation effectively lower blood lead levels, reducing pharmacological effects of lead, but they show no therapeutic benefit against morphological changes [6].

Two large, double-blind, randomized, placebo-controlled trials of chelation therapy for children found no improvement in neuropsychological function [81, 82]. However, effects of acute exposure on neurocognitive performance do show some evidence of reversibility. Removal of exposure and subsequent reduction of blood lead levels have been associated with improvements in verbal memory performance [83], visual memory performance, gross motor speed, and visual discrimination speed [84]. There is no compelling evidence for effective neurorehabilitation of deficits due to chronic lead exposure.

## 6. Discussion

Neurotoxicity involves cognitive, affective, and physiological changes caused by toxic exposure. Some of the most common toxic agents include heavy metals, and acute and chronic exposure to heavy metal Pb can induce significant neurophysiological and neuropsychological deficits, depending on the level of exposure. Consistent neuropsychological research over the years has revealed that Pb exposure can result in declines in intelligence, memory, processing speed, comprehension and reading, visuospatial skills, motor skills, and, to a probable lesser extent, executive skills. Among the cognitive deficits induced by Pb toxicity, visuospatial deficits appear to be notably prominent. Anxiety, depression, and phobia can also occur, while outcome, intervention, and rehabilitation results are largely dependent on the level of toxic exposure. There is also a growing evidence of antisocial behavior linked to early lead exposure.

Early detection, accurate assessment, and treatment are important, especially since earlier intervention may aid in reversal of certain posttoxicity sequelae. Deficits due to chronic exposure pose less favorable outcomes, making early detection and intervention that much more important. Utilization of this review's neuropsychological and neurophysiological profiles after Pb toxic exposure may aid in accurate clinical detection, intervention, assessment for tracking recovery, and policy development.
